# Multiple Molecular Mechanisms Cause Reproductive Isolation between Three Yeast Species

**DOI:** 10.1371/journal.pbio.1000432

**Published:** 2010-07-20

**Authors:** Jui-Yu Chou, Yin-Shan Hung, Kuan-Huei Lin, Hsin-Yi Lee, Jun-Yi Leu

**Affiliations:** 1 Department of Life Sciences and Institute of Genome Sciences, National Yang-Ming University, Taipei, Taiwan; 2 Institute of Molecular Biology, Academia Sinica, Taipei, Taiwan; 3 Molecular Cell Biology, Taiwan International Graduate Program, Graduate Institute of Life Sciences, National Defense Medical Center and Academia Sinica, Taipei, Taiwan; Duke University, United States of America

## Abstract

Nuclear-mitochondrial conflict (cytonuclear incompatibility) is a specific form of Dobzhansky-Muller incompatibility previously shown to cause reproductive isolation in two yeast species. Here, we identified two new incompatible genes, *MRS1* and *AIM22*, through a systematic study of F2 hybrid sterility caused by cytonuclear incompatibility in three closely related *Saccharomyces* species (*S. cerevisiae*, *S. paradoxus*, and *S. bayanus*). Mrs1 is a nuclear gene product required for splicing specific introns in the mitochondrial *COX1*, and Aim22 is a ligase encoded in the nucleus that is required for mitochondrial protein lipoylation. By comparing different species, our result suggests that the functional changes in *MRS1* are a result of coevolution with changes in the *COX1* introns. Further molecular analyses demonstrate that three nonsynonymous mutations are responsible for the functional differences of Mrs1 between these species. Functional complementation assays to determine when these incompatible genes altered their functions show a strong correlation between the sequence-based phylogeny and the evolution of cytonuclear incompatibility. Our results suggest that nuclear-mitochondrial incompatibility may represent a general mechanism of reproductive isolation during yeast evolution.

## Abstract 2

Incompatibility between nuclear and mitochondrial genomes in yeast species may represent a general mechanism of reproductive isolation during yeast evolution.

## Author Summary

Hybrids between species are usually inviable or sterile, possibly due to functional incompatibility between genes from the different species. Incompatible genes are hypothesized to encode interacting components that cannot function properly when paired with alleles from another species. To understand how incompatible gene pairs result in hybrid sterility or inviability, it is important to identify these genes and reconstruct their evolutionary history. A previous study has shown that incompatibility between nuclear and mitochondrial genomes (cytonuclear incompatibility) causes hybrid sterility between two yeast species. To expand on these findings, we screened three yeast species for genes involved in cytonuclear incompatibility, discovering two nuclear genes, *MRS1* and *AIM22*, which encode proteins that are unable to support full mitochondrial function in the hybrids. Of these two genes, Mrs1 is required for removing a specific intron in the mitochondrial *COX1* gene. By comparing different yeast species, we find a clear coevolutionary relationship between Mrs1 function and the *COX1* intron pattern. We also show that changes in three amino acids in the Mrs1 RNA-binding domain are sufficient to make Mrs1 incompatible in hybrids. Our results suggest that cytonuclear incompatibility may represent a general mechanism of reproductive isolation during yeast evolution.

## Introduction

Reproductive isolation preventing gene flow between diverging populations is crucial for the process of speciation [1]. One of the general reproductive isolation mechanisms that lead to hybrid inviability or sterility is genetic incompatibility (Dobzhansky-Muller incompatibility), which is caused by improper interactions between genetic loci that have functionally diverged in two different species [2],[3]. Since genetic incompatibility probably plays an important role at the incipient stage of speciation, identifying the incompatible loci and determining the selection forces underlying their functional divergence are vital for our understanding of how speciation occurs.

In the past two decades, scientists have discovered several genetic loci causing hybrid sterility or inviability [4],[5]. Genes involved in genetic incompatibility have been cloned from a variety of organisms, including flies, platyfishes, mice, and *Arabidopsis* [6],[7],[8],[9],[10],[11]. Nonetheless, most of the genes identified were from *Drosophila*, and in most cases, only one component of the incompatible genetic loci was cloned. Systematic studies that involve more than two species in other organisms are still rare. Baker's yeast, *Saccharomyces cerevisiae*, and its close relatives, the *Saccharomyces sensu stricto* yeasts, represent an interesting system for studying genetic incompatibility. These yeasts can mate with each other freely under laboratory conditions. Diploid hybrids collected from the wild or generated in the laboratory can reproduce asexually without showing any obvious defect. However, the viability of hybrid gametes (spores) is very low (about 0.5%–1%), suggesting that there is strong postzygotic reproductive isolation between these yeast species [12]. Because yeast differs from flies in cellular complexity, life style, and population structure, studies in yeast will greatly expand our knowledge of speciation processes [13].

Using chromosome replacement lines of hybrids of two yeast species, a previous study identified a strong incompatibility between a *S. bayanus* nuclear gene, *AEP2*, and *S. cerevisiae* mitochondria that leads to interspecific F2 hybrid sterility [14]. It was found that the 5′-UTR regions of a mitochondrion gene, *OLI1*, have diverged dramatically between these two species. Since interactions between the Aep2 protein and the 5′-UTR region of the *OLI1* mRNA are essential for *OLI1* translation, the incompatibility is probably caused by the failure of Sb-Aep2 to recognize the divergent 5′-UTR region of *Sc-OLI1*. The finding raises a few interesting questions: Does the cytonuclear incompatibility play a general role in yeast reproductive isolation, does the *AEP2-OLI1* type of interaction (activation of mRNA translation) represent a common mode of cytonuclear incompatibility, and is there a specific selective force driving this type of cytonuclear evolution?

The mitochondrion is a critical component of cellular energy production and several metabolic pathways. In many organisms including yeast, proper mitochondrial functions are required for gamete development [15],[16],[17]. The mitochondrion contains its own genome, though one in an advanced state of degeneration. Most genes essential for mitochondrial functions have been transferred from the proto-mitochondrion genome to that of their host [18],[19]. As a consequence of these events, gene products from both mitochondrial and nuclear genomes are required for proper mitochondrial operations [20],[21]. In yeast, for example, the mitochondrial genome encodes only eight proteins, but it is estimated that ∼1,000 proteins function in mitochondria [22]. Although these two genomes are under different mutation and selection pressures, they are constrained to evolve coordinately to maintain optimal functions [23]; any change in mitochondria (adaptive or drifted) may require one or more consecutive changes in the nucleus [23],[24]. This type of interaction provides an ideal background for the evolution of Dobzhansky-Muller incompatibilities; when two populations containing well-adapted cytonuclear mutations mix their genomes, unmatched mitochondria and nuclei cause reduced hybrid fitness [25].

Cytonuclear incompatibility has been observed in a wide range of organisms, including primates, amphibians, flies, wasps, a marine copepod, and a variety of plants [26],[27],[28],[29],[30],[31]. In yeast, cytonuclear incompatibility has been tested directly by transferring mitochondria from one species or strain into cells of another, which clearly showed a species barrier between mitochondrial and nuclear genomes [32],[33]. From these studies, we know that the deleterious effects of cytonuclear incompatibility can lead to reduced fitness, hybrid sterility, or inviability. Nonetheless, molecular descriptions of such an intracellular conflict are rare, and its generality as an engine of speciation remains an open question.

Here, we present results of a systematic study aimed at understanding the role of cytonuclear incompatibility in postzygotic reproductive isolation. We screened three *sensu stricto* yeasts, *S. cerevisiae*, *S. paradoxus*, and *S. bayanus* for incompatible genes causing F2 hybrid sterility and used the information about how these genes diverged in function to reconstitute the evolutionary history of the species. We found that only a few strongly incompatible gene pairs have evolved between these species. Two of them, *MRS1* and *AIM22*, were identified and the molecular mechanisms of incompatibility were characterized. By analyzing the mutations of the *MRS1* gene leading to its functional divergence between two species at the nucleotide level, we show that only three mutations make major contributions. Finally, we show that the functional divergence of these incompatible genes is correlated with the phylogeny, suggesting that cytonuclear incompatibility not only represents a general mechanism of reproductive isolation but has also occurred repeatedly during yeast evolution.

## Results

### Cytonuclear Incompatibility between Three *Saccharomyces sensu stricto* Yeasts

**Figure 1:**
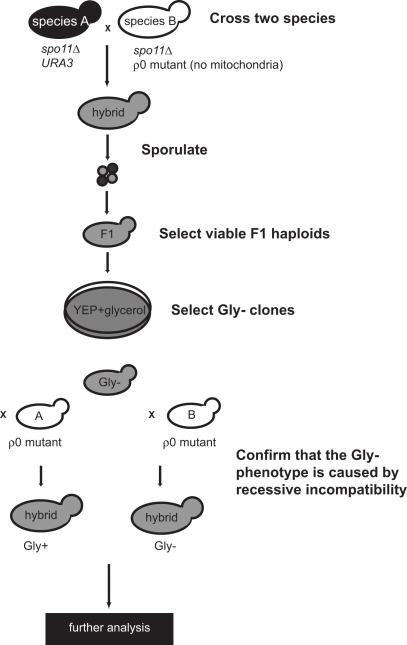
A genetic screen for hybrid haploid clones carrying incompatible nuclear-mitochondrial genomes. One species (species A) was crossed to a *ρ* ^0^ mutant of another species (species B) in which the mitochondrial DNA was completely deleted. In both strains, the *SPO11* genes were deleted to prevent meiotic recombination between homologous chromosomes. The hybrid diploids were then induced to sporulate and viable F1 spores were assayed for their ability to grow on glycerol plates (a non-fermentable carbon source). Respiration-deficient clones (Gly−) were then crossed to *ρ* ^0^ mutants of the parental strains. If the Gly− phenotype is due to loss of mtDNA, the products from both crosses will be Gly−. On the other hand, if the respiratory defect is caused by mutations occurring during the process, both products will be Gly+. Only if the Gly− phenotype is caused by recessive incompatibility will crossing with the *ρ* ^0^ mutant of species A rescue the defect (Gly+), while crossing with the *ρ* ^0^ mutant of species B will not (Gly−). The Gly− clones containing incompatible cytonuclear genomes were further analyzed to determine their chromosomal contents (see Table S1).

**Figure 2:**
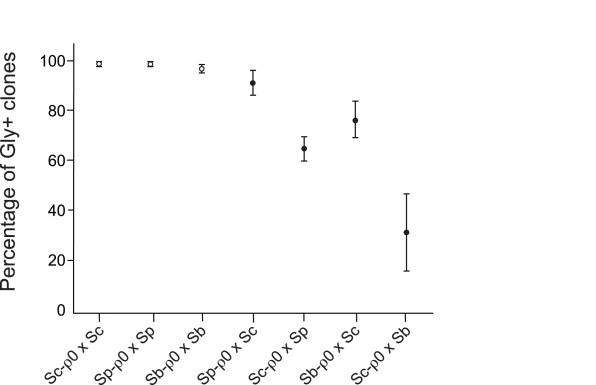
Nuclear-mitochondrial incompatibility in the hybrid F1 haploid cells. Diploid cells were constructed by crossing a respiration-proficient strain with a strain that lacked mitochondrial DNA (a *ρ* ^0^ mutant). The diploid cells were induced to sporulate and then viable haploid spores were tested for their ability to grow on glycerol plates. At least 40 viable haploid clones were checked in each experiment. Each data point represents a mean of at least three independent experiments. Open symbols represent spores from intraspecific crosses, and closed symbols represent spores from interspecific crosses. Sc, *S. cerevisiae*; Sp, *S. paradoxus*; Sb, *S. bayanus*.

A previous study has shown that incompatibility between a *S. bayanus* nuclear gene, *AEP2*, and *S. cerevisiae* mitochondria causes interspecific F2 hybrid sterility [14]. To examine whether nuclear-mitochondrial incompatibility represents a general mechanism of reproductive isolation in yeast, a systematic screen for such genes was conducted in three closely related yeast species: *S. cerevisiae* (Sc), *S. paradoxus* (Sp), and *S. bayanus* (Sb). Hybrid diploid strains between *S. cerevisiae* and *S. paradoxus*, or between *S. cerevisiae* and *S. bayanus* were induced to generate haploid spores containing different combinations of chromosomes from their parental species. These spores were then assayed for cytonuclear incompatibility. In hybrid diploids between species A and B, incompatibility could occur in two directions, between A-nucleus and B-mitochondria or between B-nucleus and A-mitochondria. When generating the yeast hybrids, we deliberately removed one parental type of mitochondria (by using *ρ* ^0^ mutants, which lack mitochondrial DNA) so that we could unambiguously assign the direction of incompatibility. After sporulation of hybrids, viable spores were measured for their ability to grow on glycerol, a non-fermentable carbon source (Figure 1). The results showed strong cytonuclear incompatibility in most of the interspecific hybrids; we observed 66%±5%, 78%±7%, and 32%±16% of respiration-proficient spores in the interspecific crosses between Sc-*ρ* ^0^ and Sp, Sb-*ρ* ^0^ and Sc, and Sc-*ρ* ^0^ and Sb, respectively, while the intraspecific crosses generated almost 100% of respiration-proficient spores (Figure 2).

The percentage of viable spores that could grow on glycerol plates was used to estimate the number of strongly incompatible loci (see Materials and Methods). The results of this growth assay suggest that there are only one or few nuclear genetic loci strongly incompatible with mitochondria in the Sc-nucleus and Sp-mitochondria, the Sb-nucleus and Sc-mitochondria, and the Sc-nucleus and Sb-mitochondria pairs. Although no strong incompatibility was detected for the Sp-nucleus and Sc-mitochondria pair, slow spore growth (on glycerol plates) was commonly observed, suggesting that some incompatibility may exist in this pair as well. Because the incompatibility between the Sb-nucleus and Sc-mitochondria has been described earlier [14], we focus here on the remaining two pairs.

### Identification of Two Cytonuclear Incompatible Genes Causing Reproductive Isolation

In our experimental design, we constructed the hybrid diploids using *spo11Δ* mutants to prevent meiotic homologous recombination. We expected that hybrid F1 haploids unable to respire (see Figure 1) should carry a specific set of chromosomes containing the incompatible genes. To identify the genes responsible for the observed cytonuclear incompatibility, the respiration-deficient clones were first examined for their chromosome contents using species-specific PCR (see Materials and Methods). In the cross between Sc-*ρ* ^0^ and Sb, the haploids carrying Sb-mitochondria but unable to respire lacked Sb-Chromosome 6, 9, or both (Table S1A). When these hybrid clones were transformed with genomic DNA libraries to screen for those genes capable of rescuing the respiratory defect, *Sb-MRS1* encoded on Sb-Chromosome 9 and *Sb-AIM22* encoded on Sb-Chromosome 6 were isolated. The results from the cross between Sc-*ρ* ^0^ and Sp are more complex, as all the respiration-deficient clones did not contain the two Sp-Chromosomes 4 and 9 (Table S1B), but were fully rescued by *Sp-MRS1* alone, which is encoded on Sp-Chromosome 9. The reason why all the hybrid clones are missing Sp-Chromosome 4 is unclear. One possibility is that Sp-Chromosome 4 is also incompatible with Sc-Chromosome 9, an issue requiring further investigation.

**Figure 3:**
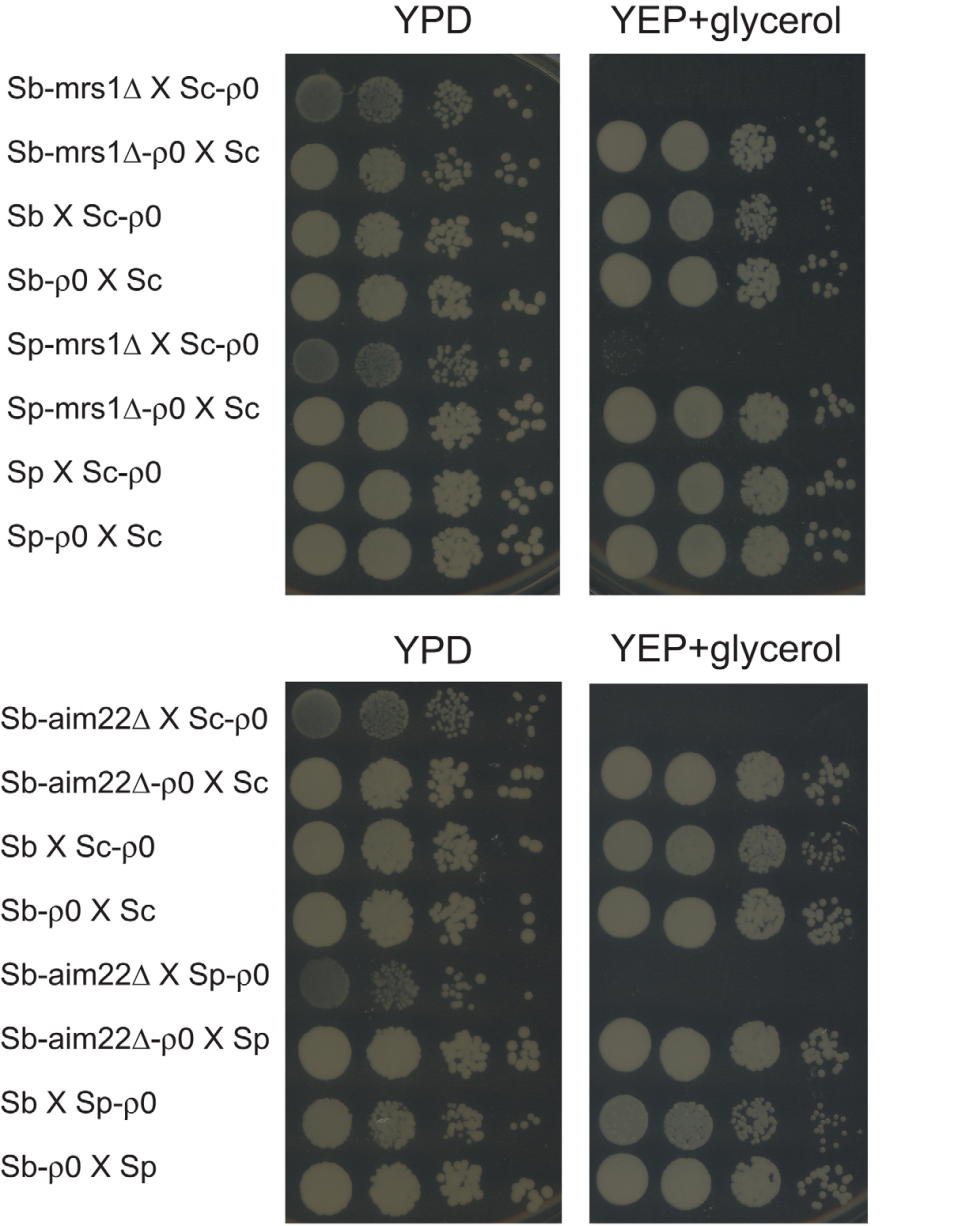
The respiratory defects caused by *Sc-MRS1* or *Sc-AIM22* are not due to nuclear-nuclear incompatibilities. Hybrid diploid cells contain two complete sets of nuclear chromosomes but only one type of mitochondrial DNA; *S. paradoxus* mitochondrial DNA in *Sp-mrs1Δ*×Sc-*ρ* ^0^ and Sp×Sc-*ρ* ^0^, *S. bayanus* mitochondrial DNA in *Sb-mrs1Δ*×Sc-*ρ* ^0^, *Sb-aim22Δ*×Sc-*ρ* ^0^ and Sb×Sc-*ρ* ^0^, and *S. cerevisiae* mitochondrial DNA in *Sp-mrs1Δ*-*ρ* ^0^×Sc, Sp-*ρ* ^0^×Sc, *Sb-mrs1Δ*-*ρ* ^0^×Sc, *Sb-aim22Δ*-*ρ* ^0^×Sc and Sb-*ρ* ^0^×Sc. The hybrid cells were serially diluted and plated on YPD or glycerol plates to measure their growth.

To rule out the possibility that the respiration defects caused by *Sc-MRS1* and *Sc-AIM22* were simply due to nuclear-nuclear incompatibility in the hybrid haploid clones, we crossed *Sp-mrs1Δ*, *Sb-mrs1Δ*, and *Sb-aim22Δ* with Sc-*ρ* ^0^ and examined the respiratory ability of the diploid cells. All the diploid cells were still respiration-deficient, even though they contained a complete set of the *S. cerevisiae* genome (Figure 3). This result demonstrated that *Sc-MRS1* is incompatible with Sp-mitochondria and that both *Sc-MRS1* and *Sc-AIM22* are incompatible with Sb-mitochondria.

Yeast cells utilize non-fermentable carbon sources to induce meiosis. Previous studies have shown that respiration-deficient cells were unable to sporulate [34]. To confirm that indeed the cytonuclear incompatibility observed in our experiments contributes to reproductive isolation, the aforementioned diploid cells (*Sp-mrs1Δ*×Sc-*ρ* ^0^, *Sb-mrs1Δ*×Sc-*ρ* ^0^, and *Sb-aim22Δ*×Sc-*ρ* ^0^) were grown on sporulation medium and examined for their sporulation efficiency. No ascus was observed in these cultures, while the control cultures sporulated efficiently. Thus, cytonuclear incompatibility caused by *Sc-MRS1* or *Sc-AIM22* results in reproductive isolation between these yeast species.

### *MRS1* Has Coevolved with the *COX1* Introns

**Figure 4:**
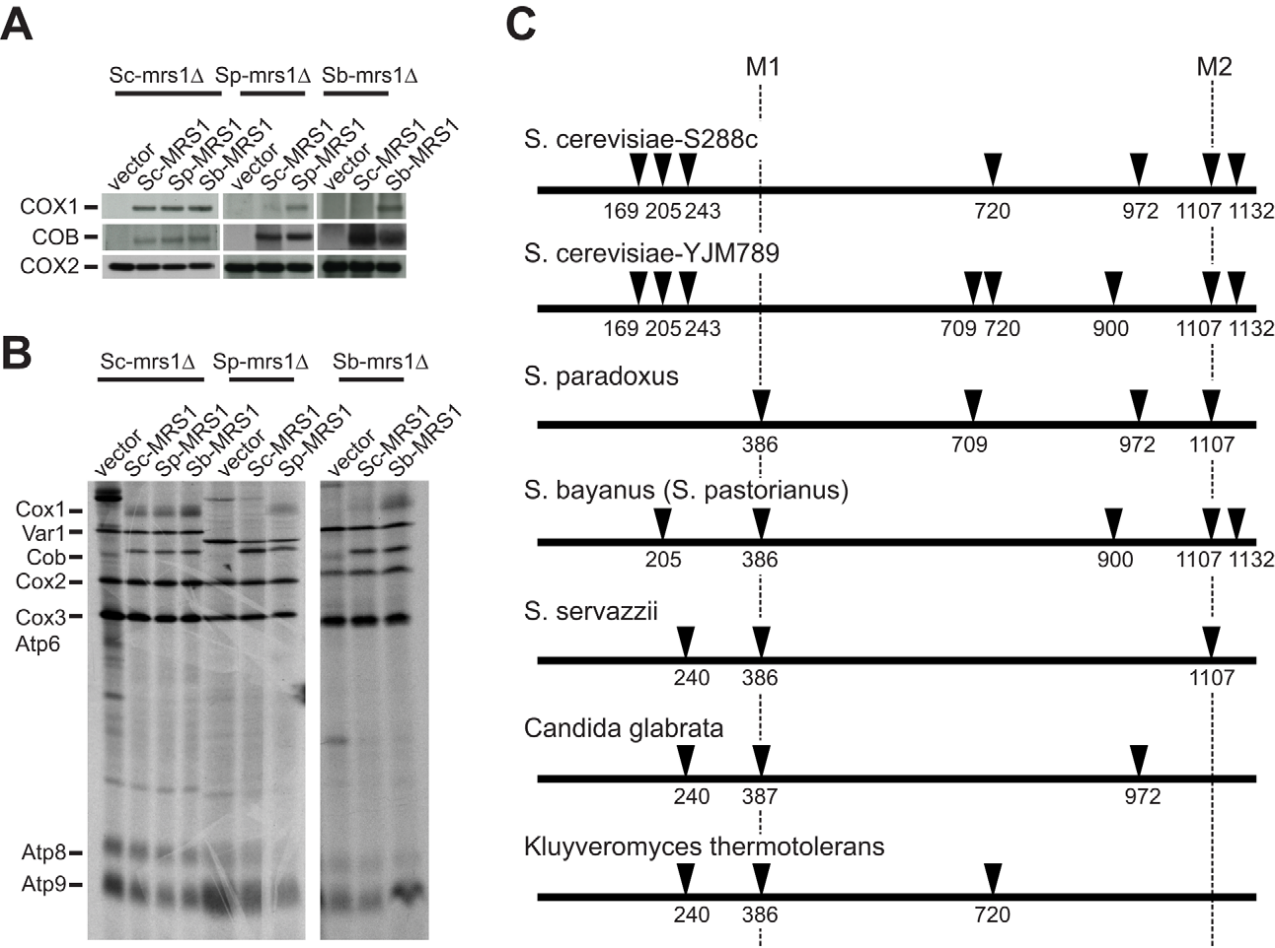
*S. cerevisiae* Mrs1 is incompatible with an ancient *COX1* intron existing in the *S. paradoxus* and *S. bayanus* mitochondrial genomes. (A) *S. paradoxus* or *S. bayanus mrs1Δ* mutants carrying *Sc-MRS1* are unable to produce mature *COX1* mRNA. Total RNA isolated from the *mrs1Δ* mutants carrying various orthologous *MRS1* genes was used for Northern blotting. *MRS1* function is involved in splicing the introns of *COX1* and *COB*, but the incompatibility caused by *Sc-MRS1* does not affect *COB* mRNA maturation. *COX2* mRNA was used as a loading control. (B) *COX1* mRNA is not translated in the *S. paradoxus* or *S. bayanus mrs1Δ* mutants carrying *Sc-MRS1*. Mitochondria were purified from the *mrs1Δ* mutants carrying various orthologous *MRS1* genes. The mitochondrial translation products were labeled with [^35^S]-methionine in the presence of cycloheximide and resolved by electrophoresis on a 17.5% SDS-polyacrylamide gel. (C) Comparison of the *COX1* introns between different yeast species. Species are arranged in order according to their phylogenetic distance from *S. cerevisiae*. Solid triangles represent introns and the numbers mark their positions in the *COX1* mRNA. M1 and M2 are the introns that require Mrs1 for their excision.

**Figure 5:**
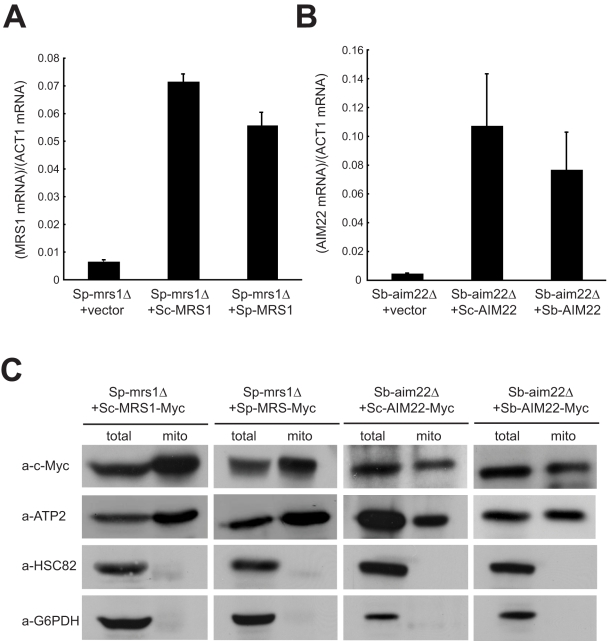
Cytonuclear incompatibility caused by *MRS1* and *AIM22* is not due to misregulation of gene transcription or failure of protein transport into mitochondria. (A) *Sc-MRS1* is transcribed at wild-type level in the *S. paradoxus mrs1Δ* mutant. Total RNA was isolated from the *S. paradoxus mrs1Δ* mutant carrying the empty vector, *Sc-MRS1* or *Sp-MRS1*, respectively, reverse transcribed, and subjected to quantitative PCR using Sp-MRS1-specific, Sc-MRS1-specific, and ACT1-specific primers. The *MRS1* mRNA levels were then normalized to the *ACT1* mRNA levels. (B) *Sc-AIM22* is transcribed at a wild-type level in the *S. bayanus aim22Δ* mutant. Total RNA was isolated from the *S. bayanus aim22Δ* mutant carrying the empty vector, *Sc-AIM22* or *Sb-AIM22*, respectively, reverse transcribed, and subjected to quantitative PCR using Sb-AIM22-specific, Sc-AIM22-specific, and ACT1-specific primers. The *AIM22* mRNA levels were then normalized to the *ACT1* mRNA levels. (C) Sc-Mrs1 localizes in the mitochondria of the *S. paradoxus mrs1Δ* mutant containing *Sc-MRS1* and Sc-Aim22 localizes in the mitochondria of the *S. bayanus aim22Δ* mutant containing *Sc-AIM22*. Mrs1 or Aim22 was fused with c-Myc at the C-terminus. Total cell extracts (total) or purified mitochondria fractions (mito) were hybridized with different antibodies to quantify the amount of proteins transported into the mitochondria. Both Hsc82 and G6PDH (glucose-6-phosphate dehydrogenase) are cytosolic proteins. Atp2 is a mitochondrial protein.

Mrs1 is a mitochondrial protein required for excision of the aI5β intron in *COX1* and the bI3 intron in *COB* [35],[36]. A previous study has shown that *S. douglasii* Mrs1 (*S. douglasii* is a synonym of *S. paradoxus*) is required to splice a *S. douglasii*-specific *COX1* intron not existing in the *S. cerevisiae COX1* [37]. We observed a similar result in *S. bayanus*. *Sc-MRS1* could not complement the respiratory defect of the *Sp-mrs1Δ* or *Sb-mrs1Δ* mutants (Figure S1). When Sb-Mrs1 or Sp-Mrs1 were replaced by Sc-Mrs1, the level of mature *COX1* mRNA was drastically reduced but the *COB* mRNA was not affected (Figure 4A). We further analyzed the translation products from purified mitochondria and confirmed that only the Cox1 protein was missing (Figure 4B). By contrast, *Sc-MRS1* transcription and protein transport into mitochondria appear to be normal in both *S. paradoxus* and *S. bayanus* (Figure 5). Thus the incompatibility between the mitochondrial and nuclear genomes is most likely due to a change in the splicing specificity of Mrs1 that occurred after *S. cerevisiae* diverged from the common ancestor of *S. cerevisiae* and *S. paradoxus*.

In order to understand how the *COX1* introns evolved in yeast, we compared *COX1* intron patterns between *S. cerevisiae*, *S. paradoxus*, *S. bayanus*, *S. servazzii*, *Candida glabrata*, and *Kluyveromyces thermotolerans*. *S. servazzii* and *C. glabrata* are species outside the *sensu stricto* complex and *K. thermotolerans* is a pre-WGD (whole-genome duplication) species. The comparison indicates that the intron in the *Sp-* or *Sb-COX1* gene incompatible with Sc-Mrs1 is an ancient intron (Figure 4C). Since this intron was eliminated only in the *S. cerevisiae* lineage, it is likely that the *Sc-MRS1* gene product lost the ability to splice this intron after the intron loss event (by adaptation or drift).

### The Functional Change of Mrs1 Is Mainly Caused by Three Nonsynonymous Mutations

**Figure 6:**
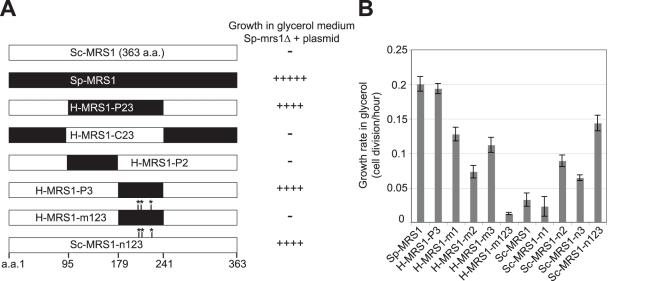
The functional change between *S. cerevisiae* and *S. paradoxus MRS1* is mainly determined by three nonsynonymous mutations. (A) Hybrid genes were constructed by fusing different regions of *Sc-MRS1* with *Sp-MRS1*. The resulting plasmid constructs were then transformed into *Sp-mrs1Δ* to test whether they could rescue its respiratory defect. The region between amino acids 179 and 241 of Sp-Mrs1 (shown in black color in H-MRS1-P3) was critical for its function in Sp-mitochondria. All nine nonsynonymous changes in this region were tested individually for their effects on the functional change. Three of them (at positions 201, 211, and 227; marked with asterisks) were found to have obvious effects. In H-MRS1-m123, these three amino acids were mutated to the *S. cerevisiae* sequences. In Sc-MRS1-n123, the same positions were mutated to the *S. paradoxus* sequences. (B) Growth rates of the *S. paradoxus mrs1Δ* mutants carrying different hybrid or mutant *MRS1* genes in glycerol-containing media. Cells were pre-adapted to the glycerol-containing medium for 1 d and those in the early log phase were used to measure the growth rate. Three independent replicates for each fitness measurement were performed. Each of H-MRS1-m1, m2, and m3 contains a single amino acid change (Sp to Sc): A201T in m1, A211V in m2, and I227M in m3, respectively. Each of Sc-MRS1-n1, n2, and n3 contains the reverse amino acid change (Sc to Sp): T201A in n1, V211A in n2, and M227I in n3, respectively.

The fact that *S. cerevisiae* and *S. paradoxus* share a high degree of nucleotide sequence identity allowed us to determine the key mutations underlying the functional change of the *MRS1* gene and to reconstruct the process of Mrs1 evolution. To this end, chimeric proteins with regions from Sc-Mrs1 and Sp-Mrs1 were constructed and assayed for their ability to complement the respiratory defect of the *Sp-mrs1Δ* mutants. We found that the functional difference between Sc-Mrs1 and Sp-Mrs1 is mainly determined by a region comprising 63 amino acids (a.a. sites 179–241; Figure 6A). Nine nonsynonymous changes have accumulated in this region since *Sc-MRS1* and *Sp-MRS1* diverged (Figure S2A). To determine which of these mutations led to the altered activity of Mrs1, we introduced the *S. paradoxus* version of each of these sites into *Sc-MRS1* and assayed their ability to rescue the *Sp-mrs1Δ* respiratory defect. Analogous experiments were performed in the reverse direction by introducing Sc-specific amino acids into *Sp-MRS1*. Only mutations in three amino acids (Sc to Sp: T201A, V211A, and M227I) had obvious contributions (Figure 6B and Figure S2). When all three mutations were combined together in a single mutant clone, it explained most of the effect. The growth rate of cells carrying the mutant plasmid (Sc-MRS1-n123) in a glycerol-containing medium is about 75% of that of wild type cells. Interestingly, these three amino acids are all conserved in *S. paradoxus*, *S. kudriavzevii*, and *S. bayanus* but are changed in *S. cerevisiae*. Among the other six nonsynonymous changes in this region, only two of them (Sc to Sp: K186E and R223Q) share the same pattern. This observation is consistent with our hypothesis that the functional change of *MRS1* occurred only after an ancestral *COX1* intron was lost in *S. cerevisiae*. Our results clearly demonstrate that the observed nuclear-mitochondrial incompatibility results from cumulative effects of multiple mutations. On the other hand, they also suggest that only a small fraction of the nonsynonymous changes between species contributes to the incompatibility.

The Mrs1 protein does not contain any specific functional domain. However, a recent study has used computer modeling to predict the Mrs1 protein structure [38]. We examined the relative positions of these residues using the predicted structure. All three residues (a.a. 201, 211, and 227) were found to localize on the RNA-binding surface (Figure S3). It is possible that these amino acid changes have altered the substrate specificity of Mrs1 that leads to the incompatibility.

### *Sc-AIM22* Is Not Compatible with Sb-Mitochondria

*S. cerevisiae* nuclei can only support *S. bayanus* mitochondria if the cells contain a *S. bayanus AIM22* gene. *AIM22* encodes a lipoate-protein ligase homologous to the bacterial *lplA* protein [39],[40]. In eukaryotic cells, lipoic acid has been shown to be an essential cofactor to a variety of mitochondrial proteins and lipoate-protein ligase (together with other enzymes) is required to lipoylate these mitochondrial targets [39],[41]. We have not investigated which mitochondrial protein or enzyme in Sb-mitochondria is incompatible with Sc-Aim22. However, our data indicate that the incompatibility is not caused by misregulation of the *Sc-AIM22* transcription or failure to transport the Sc-Aim22 to Sb-mitochondria (Figure 5).

To investigate whether the *AIM22* gene in the *S. cerevisiae-S. paradoxus* branch has been under positive selection during evolution, we measured the ratio of nonsynonymous (Ka) to synonymous (Ks) nucleotide substitution rates between these species. The Ka/Ks values of *AIM22* in the *S. cerevisiae–S. paradoxus*, *S. cerevisiae–S. bayanus*, and *S. paradoxus–S. bayanus* pairs are 0.13, 0.12, and 0.14, showing no sign of positive selection (Ka/Ks>1). We also ran a PAML's branch-model analysis on the *AIM22* gene [42],[43] but could not detect any signature of significant positive selection (Figure S4).

Previous studies of *AEP2* have shown that the nuclear-mitochondrial incompatibility is asymmetrical. While Sb-Aep2 is completely incompatible with Sc-mitochondria, Sc-Aep2 retains partial compatibility with Sb-mitochondria [14]. We also found that incompatibility caused by *AIM22* and *MRS1* only occurred in one direction. Although *Sc-AIM22* and *Sc-MRS1* are not compatible with Sb-mitochondria, *Sb-AIM22* could complement the *Sc-aim22Δ* mutant and both *Sb-* and *Sp-MRS1* could rescue the *Sc-mrs1Δ* mutant.

### The Evolution of Cytonuclear Incompatibility Is Correlated with the Phylogeny

**Figure 7:**
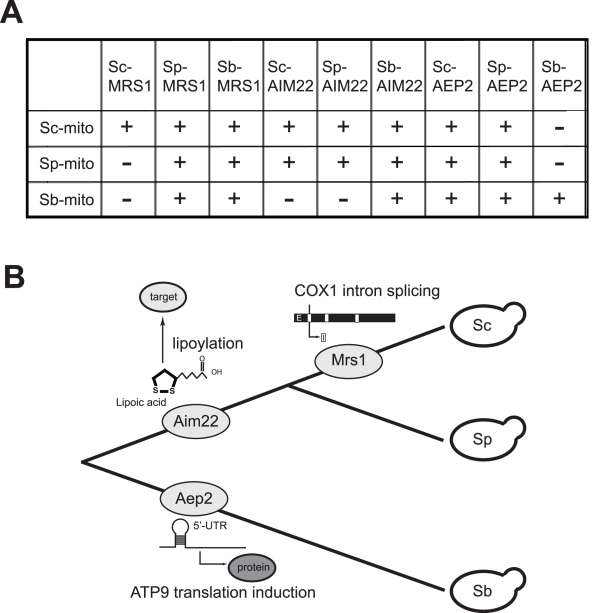
The evolution of incompatibility is correlated with the phylogeny. (A) Results of the functional complementation assay indicate that the functional change of Mrs1, Aim22, and Aep2 occurred at different periods of time. Different orthologous alleles of *MRS1*, *AIM22*, or *AEP2* were transformed into *S. cerevisiae*, *S. paradoxus*, or *S. bayanus* mutants in which the wild-type copy had been deleted. The transformants were then tested for their ability to grow on glycerol plates. If the transformant was able to grow on the glycerol plate, it suggested that the mitochondria were compatible with the gene (which is indicated by the plus sign). Sc-mito, Sc-mitochondria; Sp-mito, Sp-mitochondria; Sb-mito, Sb-mitochondria. (B) A diagram of the phylogeny of three closely related *Saccharomyces* species showing the branches in which functional changes of the cytonuclear incompatible genes have occurred and the molecular functions of these genes.

Nuclear-mitochondrial incompatibility has been shown to occur commonly between different yeast species [32],[33]. However, it is unclear whether nuclear-mitochondrial incompatibility between different pairs of species has evolved at different periods of time in different species lineages. To address this issue, we tested the compatibility between different orthologues of these incompatible genes and mitochondria from each species. Different orthologous alleles of *MRS1*, *AIM22*, or *AEP2* were transformed into the *S. cerevisiae*, *S. paradoxus*, or *S. bayanus* mutants in which the wild-type copy had been deleted. The transformants were then tested for their ability to grow on glycerol plates (Figure 7A and Figure S1). Information from these assays was used to deduce the time of occurrence of the functional change leading to incompatibility. A clear correlation between the emergence of cytonuclear incompatibility and the phylogeny is observed (Figure 7B). *Sc-MRS1* is incompatible with Sp-mitochondria or Sb-mitochondria, indicating that the functional change of *Sc-MRS1* occurred only in the *S. cerevisiae* lineage. On the other hand, the functional change of *AIM22* represents a more ancient event in the common ancestor of *S. cerevisiae* and *S. paradoxus*, because *Sc-AIM22* and *Sp-AIM22* are exchangeable but neither of them is compatible with Sb-mitochondria. Finally, the data suggest that *Sb-AEP2* diverged in function only in the *S. bayanus* lineage since *Sb-AEP2* is incompatible with either Sc-mitochondria or Sp-mitochondria. These results provide evidence that nuclear-mitochondrial incompatibility has repeatedly arisen during the history of yeast evolution and probably represents an important reproductive isolation mechanism in yeast species.

## Discussion

Previous studies in *Saccharomyces* yeasts have suggested that the deleterious effect of DNA sequence divergence on meiotic recombination probably contributes in part to reproductive isolation during yeast evolution [12],[44],[45]. Our results, on the other hand, suggest that nuclear-mitochondrial incompatibility is also a promising candidate for causing intrinsic hybrid dysfunction. In fact, these two mechanisms are not mutually exclusive. Genomes from different populations will accumulate enough DNA sequence divergence only after extended periods of allopatric isolation, but the effect of sequence divergence can be applied directly in the diploid F1 hybrid cells. Cytonuclear incompatibility can be achieved by only a few mutations, and its deleterious effect can be carried on to the F1 gamete or F2 progeny. In theory, cytonuclear incompatibility has a stronger impact on blocking gene flow between populations in the early stages of speciation, and reproductive isolation is reinforced later on when populations have accumulated enough DNA sequence divergence. These two mechanisms can be complementary to each other in terms of their effects and evolutionary trajectories. It will be interesting to investigate whether cytonuclear incompatibility exists between different populations of the same species.

Reproductive isolation resulting from genetic incompatibility has been discovered in a variety of organisms [7]. Most of the examples characterized so far are caused by interactions between nuclear genes. In yeast, this type of incompatibility has been investigated in a few studies, yet no strongly nuclear-nuclear incompatible genes were identified [14],[46]. On the other hand, cytonuclear incompatibilities were observed in hybrids between different yeast species or populations [47]. Cytonuclear incompatibility probably represents a more general mechanism of reproductive isolation in yeast. By analyzing the functions and the interacting components of the identified incompatible genes, we discovered that cytonuclear incompatibility could be achieved by multiple molecular mechanisms: intron splicing, protein lipoylation, and activation of mRNA translation. This suggests that cytonuclear incompatibility in yeast can occur in various pathways by diverse molecular mechanisms.

Scientists usually compare orthologous sequences from different species and use the detected molecular signatures to infer the evolutionary process of a gene. Since our data suggest that Mrs1 changed function only after *S. cerevisiae* diverged from *S. paradoxus*, it is reasonable to assume that the altered function originated from amino acid changes that occurred specifically in the *S. cerevisiae* lineage. By comparing the coding regions of the *MRS1* orthologues from *S. cerevisiae*, *S. paradoxus*, *S. kudriavzevii*, and *S. bayanus*, we observed 22 amino acids that are different in *S. cerevisiae* but are conserved in the other three species. Interestingly, our functional assays showed that only three of these amino acid changes contribute significantly to the functional differences of Mrs1. The other mutations may have very minor effects unable to be detected by our functional assays or have been fixed simply by genetic drift. In a previous study, Rawson and Burton have also observed that three amino acid changes in a nucleus-encoded cytochrome c (CYC) are responsible for cytonuclear incompatibility between different populations of a marine copepod, *Tigriopus californicus* [48]. These results illustrate the importance of mapping the critical amino acid changes in order to understand how a gene evolved.

What is the major driving force underlying the evolution of mitochondria? It is known that the mitochondrial genome suffers a higher mutation load because it is constantly facing higher levels of oxidative reagents and its DNA protection system is more primitive as compared to the nuclear genome. With a much smaller copy number of mitochondrial DNA in yeast (30–80 molecules of mitochondrial genomes in yeast cells compared to 1,000–5,000 molecules in animal cells), mutations may be fixed frequently in the mitochondrial genome by genetic drift. Since wild yeast often propagate clonally in natural environments [13], a founder cell with mild deleterious mutations in its mitochondrial genome may have a chance to accumulate suppressors in the mitochondrial or nuclear genomes to rescue the fitness before its progeny are outcompeted by cells from another population. Alternatively, mitochondrial evolution may be driven by an “arms race” process between selfish mitochondrial DNA and the “wild-type” mitochondrial or nuclear genomes. It is commonly observed in yeast that by manipulating the host replication or segregation systems, some mitochondrial genomes allow themselves to be inherited more efficiently, even though they may be carrying compromised respiratory functions [49]. In a sexual population, the other mitochondrial or host nuclear genomes will be selected to counteract this selfish behavior or the deleterious effects carried by the selfish DNA. Such an “arms race” may allow mitochondrial genomes to evolve faster than by genetic drift (in a fashion similar to positive selection). Incompatibilities driven by arms races between different genetic components have been suggested in a few recent studies [7]. Among these genetic conflicts, most of them are caused by selfish elements manipulating segregation distortion [11],[50],[51],[52],[53]. The “hybrid necrosis” phenotype observed in *Arabidopsis* probably results from recurrent conflicts between the host defense system and pathogens [10]. The genetic conflict caused by selfish mitochondrial genomes may represent another type of arms race. It will be interesting to investigate whether the arms race model can explain the cytonuclear incompatibilities observed in other organisms. Finally, ecological adaptation may also contribute to mitochondrial evolution. Evidence suggests that adaptive mutations have occurred in mitochondria in response to different environmental stresses that interfere with cellular energy demands [48],[54],[55]. In yeast, it has been shown that *S. bayanus* grows much better than *S. cerevisiae* on media containing only non-fermentable carbon sources, with the opposite observed in fermentable media. It has been speculated that the changes in *Sb-AEP2* and *Sb-OLI1* are a part of such ecological adaptation [14].

Reciprocal crosses between species often generate asymmetrical hybrid viability or sterility, a general feature of intrinsic postzygotic isolation called Darwin's corollary [56],[57],[58],[59],[60]. The Dobzhansky-Muller model suggests that alleles causing reproductive isolation act asymmetrically [61]. However, asymmetries in allele action do not necessarily lead to asymmetries in reproductive isolation [62]. Incompatibility between autosomal loci affects both reciprocal crosses identically. Asymmetric reproductive isolation is usually caused by incompatibility between autosomal loci and uniparentally inherited materials, such as cytoplasmic elements and sex chromosomes. It has been suggested that cytonuclear incompatibility caused by different trajectories of mitochondrial evolution in different species may contribute to this phenomenon [59],[63]. Our results provide the molecular basis to support this hypothesis. Since cytonuclear incompatibility can be achieved by multiple molecular mechanisms and evolve at different rates in different lineages, it can serve as a general mechanism of reproductive isolation and also create asymmetrical reproductive isolation between species.

## Materials and Methods

### Strains and Genetic Procedures

Yeast strain genotypes are listed in Table S2. The parental *S. cerevisiae* strains (JYL1127 and JYL1128) are isogenic with *W303* (*MATa ura3-1 his3-11,15 leu2-3,112 trp1-1 ade2-1 can1-100*). The parental *S. paradoxus* strains (JYL1137 and JYL1138) are derived from YDG 197 and are a gift from Dr. Duncan Greig (University College London, UK). The parental *S. bayanus* strains (JYL1030 and JYL1031) were derived from a strain (*S. bayanus* #180) collected by Dr. Duccio Cavalieri (University of Florence, Italy). The strains JYL1157, 917, and 1256 were used for measuring hybrid fertility. Substitutive and integrative transformations were carried out by the lithium acetate procedure [64]. Media, microbial, and genetic techniques were as described [65].

### Estimation of the Nuclear-Mitochondrial Incompatible Gene Number between Different Species

a and α cells of one species (*S. cerevisiae*, *S. paradoxus*, *or S. bayanus*) were crossed to α and a *ρ* ^0^ cells of another species to generate F1 hybrid cells. In both species, the *SPO11* genes were deleted to prevent meiotic recombination between homologous chromosomes. Strains from the first species also had a *URA3* marker inserted near the centromere of a chromosome so that haploid spores could be efficiently selected on 5-FOA plates. After the hybrid diploids were induced to sporulate, viable spores were tested for their respiratory ability. Respiration-deficient clones were further crossed with *ρ* ^0^ mutants of the parental species to confirm that the defect was caused by cytonuclear genomic incompatibility (Figure 1). A genetic analysis was used to estimate the number of the nuclear genes that are incompatible with mitochondrial DNA from another species: in a cross between two *spo11Δ* mutants, meiotic recombination does not occur and homologous chromosomes segregate randomly [66],[67]. For a specific chromosome A, 25% of the spores will carry two A chromosomes (of both parental types), 50% of them will carry one A chromosome, and 25% of them will have no A chromosome (these cells will not survive so they will not be counted in our later analysis). If only one gene on chromosome A is recessively incompatible with mitochondrial DNA (in our case, all spores from a single cross carry the same parental type of mitochondrial DNA), we expect to see that 66% of the viable spores are Gly+ (1/3+2/3×1/2 = 66%). The spores carrying two A chromosomes should be Gly+ because the incompatibility is recessive. Half of the spores that carry only one A chromosome should be Gly+ if their A chromosome is from the same parent of the mitochondrial DNA. If two genes on different chromosomes are involved, we expect to see that 43% of the viable spores are Gly+ (66%×66% = 43%). Epistatic effects are not taken into account in this analysis because considering such effects would make it impossible to estimate the involved locus number.

### Genetic Screens and Genotyping of the Hybrid Haploids with Incompatible Nuclear-Mitochondrial Genomes

We examined the chromosome composition of respiration-deficient hybrid lines by PCR using species-specific primers for all 16 chromosomes. Yeast genomic DNA libraries constructed from *S. bayanus* or *S. paradoxus* genomes were transformed into the Gly− clones to screen for incompatible genes.

### RNA Isolation and Northern Analyses of Mitochondrial Transcripts

Yeast strains were grown in 3 ml YPD liquid cultures at 30°C to stationary phase and total RNA was isolated using Qiagen RNeasy Midi Kits (Qiagen, Valencia, CA). Ten µg of total RNA was separated on a 1.3% agarose-formaldehyde gel and then transferred to a nylon membrane (Millipore, Billerica, MA). Northern blotting was performed as described [68]. Because both *COX1* and *COB* signals were difficult to be completely washed out and the background was high after the second hybridization, we used the same RNA samples to load three repeats on the same gel, cut the membrane after transferring, and hybridized each repeat with one specific probe. The gene-specific primers for the DIG-labeled probes were described in Rodeheffer et al. [69] except for the probe of *COX2*. The primers for *COX2* were 5′-TTAATGATAGTGGTGAAACTGTTG-3′ and 5′-CCAAAGAATCAAAATAAATGCTCG-3′. The probe generation and hybridization were as described in the Genius System User's Guide (Roche, Indianapolis, IN).

### Quantitative PCR

Total RNA was isolated using Qiagen RNeasy Midi Kit (Qiagen). First-strand cDNA was synthesized using High Capacity cDNA Reverse Transcriptase Kit (Applied Biosystems, Foster City, CA) at 37°C for 2 h. A 25-fold dilution of the reaction products was then subjected to real-time quantitative PCR analysis using gene-specific primers, the SYBR Green PCR master mix, and an ABI-7000 sequence detection system (Applied Biosystems). Data were analyzed using the built-in analysis program.

### Isolation of Mitochondria and Western Blot Analyses

Yeast cells were cultured in selection medium at 30°C to the mid-exponential phase. Mitochondria fractions were prepared as described [70]. The post-mitochondrial supernatant (PMS) fractions were collected immediately following centrifugation of mitochondria. The PMS was precipitated with ice-cold 10% trichloroacetic acid (TCA) and centrifuged at 14K rpm for 15 min at 4°C, followed by an ice-cold acetone wash using the same centrifugation conditions. Laemmli sample buffer was added to the mitochondrial pellets resuspended in TE buffer plus protease inhibitors, and to the PMS fractions. For Western blot analyses, mitochondrial protein (40 µg) was separated by SDS-PAGE using the GE Healthcare Life Science system (Mini-Vertical Units SE260) and then transferred to a BioRad Immun-Blot PVDF membrane in transfer buffer (25 mM Tris base, 200 mM glycine, 20% methanol, 0.01% SDS). The immunopositive bands were visualized by using Western Lightning chemiluminescence reagent (PerkinElmer, Waltham, MA). Anti-G6PDH polyclonal antibody was purchased from Sigma (St. Louis, MO). Anti-c-Myc polyclonal antibody (A-14) was from Santa Cruz Biotechnology (Santa Cruz, CA). Anti-Hsc82 and Anti-Atp2 polyclonal antibodies were obtained from Dr. Chung Wang (Academia Sinica, Taipei).

### In Vivo Radiolabeling of Mitochondrial Proteins

Cells in the early log phase were inoculated in 2 ml standard minimal medium and grown for 30 min. Cycloheximide stock solution (10 ml/mg in dH_2_O) was added to a final concentration of 100 µg/ml. Cells were incubated for 5 min prior to addition of 0.1 mCi of [^35^S]-methionine. The reaction was terminated after 1 h by adding 2 ml of chase solution (1% casamino acid, 2 mg/ml Na_2_SO_4_). Mitochondria were prepared as described [65]. The radiolabeled proteins were separated on a 17.5% polyacrylamide gel.

### Prediction of the Ancestral *AIM22* Sequence

The ancestral *AIM22* sequence of *S. cerevisiae* and *S. paradoxus* was constructed using a maximum likelihood procedure (free-ratio model) as implemented in the PAML package. The Ka/Ks ratios were calculated using DNAsp 5.0 [71].

### Branch-Model Analysis for *AIM22*

The Ka/Ks ratios were estimated using the free-ratio branch model of PAML. This method allows the Ka/Ks ratios to vary among branches in a given phylogeny and is useful in detecting positive selection acting on particular lineages [42]. The *AIM22* sequences from five *Saccharomyces sensu stricto* species (*S. cerevisiae*, *S. paradoxus*, *S. mikatae*, *S. bayanus*, and *S. kudriavzevii*) were used in our analysis. Results of the branch-model PAML analysis suggested that no particular lineage was subjected to positive selection (i.e., Ka/Ks>1) (Figure S4). Because both *Sc-AIM22* and *Sp-AIM22* are incompatible to *S. bayanus* mitochondria, it is possible that there has been an accelerated sequence evolution in either the lineage leading to *S. cerevisiae* and *S. paradoxus*, or in the lineage leading to *S. bayanus*. To test this hypothesis, we performed an analysis as described in Yang and Nielsen [43]. One ω-ratio and two ω-ratio branch models were implemented. The first model assumes that only one ω-ratio leads to whole phylogeny branches and the second model assumes that one ω-ratio leads to the branch that we are interested in and another ω-ratio leads to the rest of the other branches. Twice the difference of their likelihood ratio between any two models (likelihood ratio test; LRT) was then compared against a chi-square distribution. The degree of freedom (d.f.) was obtained based on the difference of parameters used in two different models. From our analysis, no accelerated evolution was observed in any lineage along the phylogeny of *AIM22*.

## Supporting Information

Figure S1 **Functional complementation assays of different orthologous** ***MRS1*** **and** ***AIM22*** **genes.** Orthologous *MRS1* and *AIM22* genes from different species were cloned into single-copy plasmids and then transformed into the *S. cerevisiae*, *S. paradoxus*, or *S. bayanus* mutants in which the wild-type copy had been deleted. The transformants were serially diluted and plated on YPD or glycerol plates to measure their growth. The empty vector was used as a control in the experiment.
(3.80 MB TIF)

Figure S2 **Growth rates of the** ***S. paradoxus mrs1Δ*** **mutants carrying different hybrid or mutant** ***MRS1*** **genes in glycerol-containing media.** (A) Protein sequence alignment between Sp-Mrs1 and Sc-Mrs1. Only the region between amino acids 177 and 241 is shown. The numbers indicate positions of the nonsynonymous changes. (B) Growth rates of the *S. paradoxus mrs1Δ* mutants carrying different mutant *Sc-MRS1* genes in glycerol-containing media. Each of *Sc-MRS1* mutants contains a single amino acid change (from Sc to Sp). (C) Growth rates of the *S. paradoxus mrs1Δ* mutants carrying different mutant *H-MRS1* genes in glycerol-containing media. Each of *H-MRS1* mutants contains a single amino acid change (from Sp to Sc). Cells were pre-adapted to the glycerol-containing medium for 1 d and those in the early log phase were used to measure the growth rate. Three independent replicates for each fitness measurement were performed.
(0.50 MB EPS)

Figure S3 **The critical nonsynonymous changes occur at the residues positioned on the RNA-binding surface.** A structural model for the Mrs1 protein-RNA complex. The RNA backbone is shown as a tube and the domains protected by Mrs1 binding are red. Mrs1 dimers are colored dark and light gray. The three critical amino acids with nonsynonymous substitutions (201, 211, and 227) are shown as red spheres on the Mrs1 proteins.
(0.92 MB EPS)

Figure S4 **Evolutionary rate analysis of** ***AIM22*** **from different** ***Saccharomyces*** **yeast species.** Dendrogram showing phylogenetic relationships among related *Saccharomyces* yeast species. (A) The Ka/Ks ratios for the entire *AIM22* coding sequence in different *Saccharomyces* lineages. The tree branch length and branch-wise Ka/Ks ratio were estimated using a free-ratio model by Codeml in the PAML package. (B). The lineage that leads to *S. cerevisiae* and *S. paradoxus* was treated as foreground branches (ω2) and the other as background branches (ω1). When ω2 was compared with ω1, no significantly accelerated rate was detected using LRT (*p* = 0.5902). (C). The lineage that leads to *S. bayanus* was treated as foreground branches (ω2) and the other as background branches (ω1). No significantly accelerated rate was detected between these two branches (*p* = 0.1407). 2δl, twice the difference of likelihood values from two models; d.f., degree of freedom; H_0_, null model; H_1_, alternative model.
(0.66 MB EPS)

Table S1 **Chromosome genotyping of the hybrid haploids with deficient mitochondrial functions.** Genomic DNAs isolated from the F1 Gly− haploid cells (Figure 1) were analyzed by PCR with species-specific primers for each chromosome (+, chromosome detected). After *MRS1* and *AIM22* were identified to cause the cytonuclear incompatibility, plasmids carrying *Sb-MRS1*, *Sb-AIM22*, *Sb-MRS1-AIM22*, or *Sp-MRS1* were transformed into each clone to see whether the respiratory defect could be rescued (v, rescued; x, not rescued; nd, not determined).
(0.26 MB DOC)

Table S2 **Yeast strains.**
(0.04 MB DOC)
