# Supplementary figures and images for: Multiple Molecular Mechanisms Cause Reproductive Isolation between Three Yeast Species
Source: PLoS Biol. 2010 Jul 20;8(7):e1000432. doi: 10.1371/journal.pbio.1000432 (PMC2907292; doi:10.1371/journal.pbio.1000432)

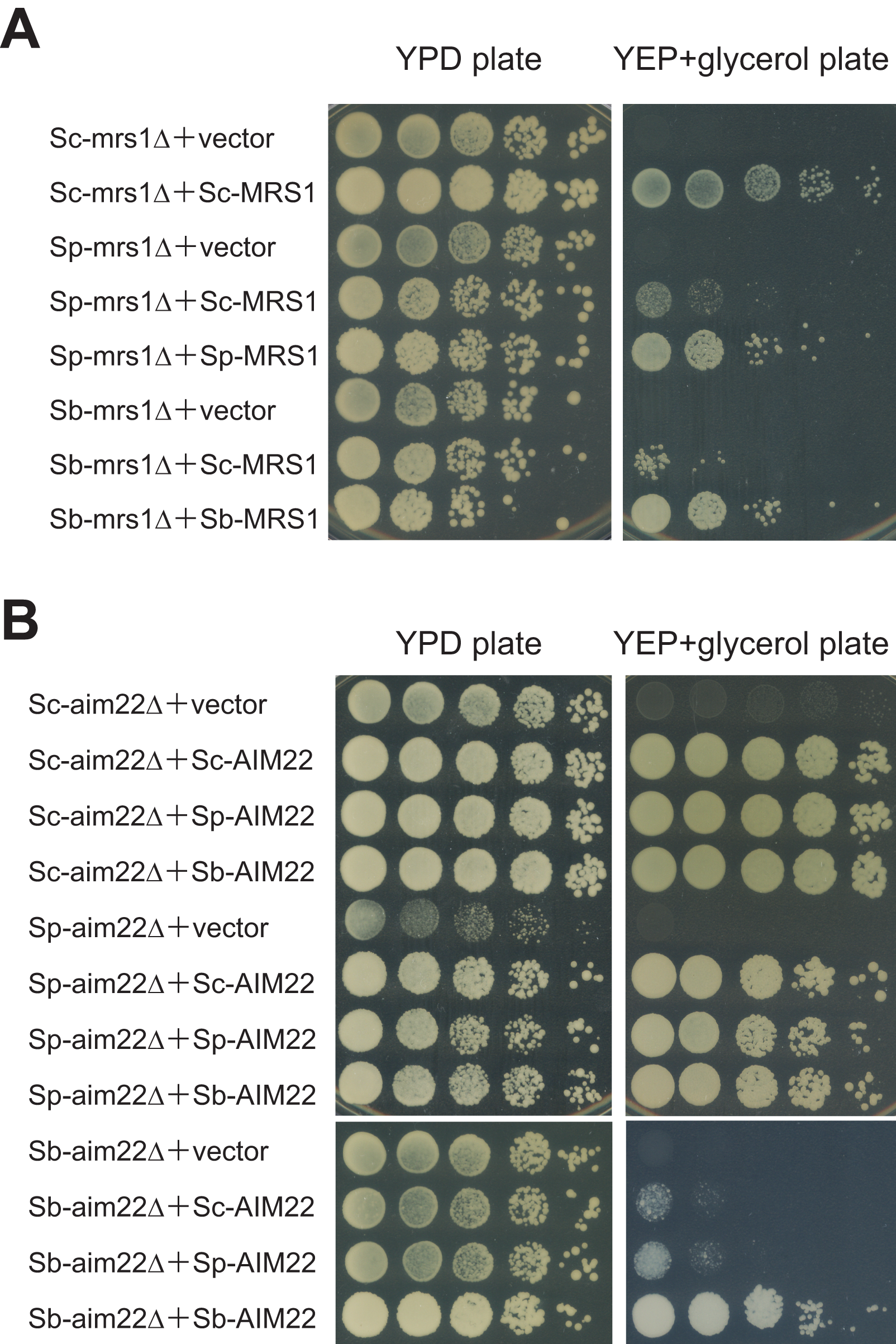

Supplement: Figure S1 — Functional complementation assays of different orthologous MRS1 and AIM22 genes. Orthologous MRS1 and AIM22 genes from different species were cloned into single-copy plasmids and then transformed into the S. cerevisiae, S. paradoxus, or S. bayanus mutants in which the wild-type copy had been deleted. The transformants were serially diluted and plated on YPD or glycerol plates to measure their growth. The empty vector was used as a control in the experiment. (3.80 MB TIF) [file pbio.1000432.s001.tif]
