# Supplementary material for: Multiple Molecular Mechanisms Cause Reproductive Isolation between Three Yeast Species
Source: PLoS Biol. 2010 Jul 20;8(7):e1000432. doi: 10.1371/journal.pbio.1000432 (PMC2907292; doi:10.1371/journal.pbio.1000432)
Supplement: Table S1 — Chromosome genotyping of the hybrid haploids with deficient mitochondrial functions. Genomic DNAs isolated from the F1 Gly− haploid cells (Figure 1) were analyzed by PCR with species-specific primers for each chromosome (+, chromosome detected). After MRS1 and AIM22 were identified to cause the cytonuclear incompatibility, plasmids carrying Sb-MRS1, Sb-AIM22, Sb-MRS1-AIM22, or Sp-MRS1 were transformed into each clone to see whether the respiratory defect could be rescued (v, rescued; x, not rescued; nd, not determined). (0.26 MB DOC) [file pbio.1000432.s005.doc]

Table S1A. Hybrid clones from a cross between *S. cerevisiae* and *S. bayanus*

| *Clone#* | *Sb-Chromosome* | | | | | | | | | | | | | | | | *Sb-MRS1* | *Sb-AIM22* | *Sb-MRS1 + AIM22* |
| --- | --- | --- | --- | --- | --- | --- | --- | --- | --- | --- | --- | --- | --- | --- | --- | --- | --- | --- | --- |
|  | *1* | *2* | *3* | *4* | *5* | *6* | *7* | *8* | *9* | *10* | *11* | *12* | *13* | *14* | *15* | *16* |  |  |  |
| *YML56* |  | *+* |  | *+* |  |  | *+* |  | *+* |  |  |  | *+* | *+* | *+* |  | *x* | *v* | *v* |
| *YML69* |  |  |  | *+* | *+* |  | *+* |  | *+* |  |  |  | *+* |  |  | *+* | *x* | *v* | *v* |
| *YML14* | *+* |  | *+* | *+* | *+* |  | *+* | *+* | *+* |  |  | *+* | *+* | *+* | *+* |  | *x* | *v* | *v* |
| *YML30* | *+* |  | *+* | *+* | *+* |  |  | *+* | *+* | *+* |  |  | *+* | *+* | *+* | *+* | *x* | *v* | *v* |
| *YIL13* | *+* |  | *+* | *+* | *+* |  |  | *+* | *+* |  |  | *+* |  | *+* | *+* |  | *x* | *v* | *v* |
| *YML31* | *+* |  | *+* | *+* | *+* |  |  |  | *+* | *+* |  | *+* | *+* |  |  | *+* | *x* | *v* | *v* |
| *YML65* | *+* |  | *+* | *+* |  |  | *+* | *+* | *+* |  | *+* | *+* | *+* | *+* | *+* |  | *x* | *v* | *v* |
| *YML66* | *+* |  | *+* | *+* |  |  | *+* |  | *+* | *+* |  | *+* | *+* |  |  | *+* | *x* | *v* | *v* |
| *YEL1* | *+* |  |  | *+* | *+* |  | *+* | *+* | *+* |  | *+* |  |  | *+* | *+* |  | *x* | *v* | *v* |
| *YIL12* | *+* |  |  | *+* | *+* |  |  |  | *+* | *+* |  |  | *+* |  |  |  | *x* | *v* | *v* |
| *YML5* | *+* |  |  | *+* | *+* |  |  |  | *+* |  |  |  | *+* |  |  |  | *x* | *v* | *v* |
| *YML61* | *+* | *+* |  | *+* | *+* |  | *+* | *+* | *+* |  |  |  | *+* |  | *+* | *+* | *x* | *v* | *v* |
|  |  |  |  |  |  |  |  |  |  |  |  |  |  |  |  |  |  |  |  |
| *YML62* |  |  | *+* |  |  | *+* | *+* |  |  | *+* | *+* | *+* | *+* |  |  | *+* | *v* | *x* | *v* |
| *YEL16* | *+* |  |  |  | *+* | *+* | *+* |  |  | *+* |  |  | *+* |  |  |  | *v* | *x* | *v* |
| *YML47* | *+* | *+* |  |  | *+* | *+* | *+* |  |  | *+* |  |  | *+* |  | *+* |  | *v* | *x* | *v* |
| *YML49* | *+* | *+* | *+* | *+* |  | *+* | *+* | *+* |  | *+* | *+* |  | *+* |  | *+* |  | *v* | *x* | *v* |
| *YML26* | *+* | *+* | *+* | *+* |  | *+* | *+* | *+* |  | *+* |  | *+* | *+* |  | *+* | *+* | *v* | *x* | *v* |
|  |  |  |  |  |  |  |  |  |  |  |  |  |  |  |  |  |  |  |  |
| *YML29* |  | *+* | *+* |  | *+* |  |  | *+* |  |  |  | *+* | *+* |  |  | *+* | *x* | *x* | *v* |
| *YML53* |  |  | *+* |  | *+* |  | *+* | *+* |  |  | *+* |  | *+* |  |  | *+* | *x* | *x* | *v* |
| *YML67* |  |  | *+* |  |  |  |  |  |  | *+* |  | *+* | *+* |  |  |  | *x* | *x* | *v* |
| *YML59* | *+* |  | *+* |  | *+* |  | *+* |  |  |  |  | *+* | *+* |  |  | *+* | *x* | *x* | *v* |
| *YML33* | *+* |  | *+* |  |  |  |  |  |  |  |  | *+* | *+* |  |  | *+* | *x* | *x* | *v* |
| *YEL5* | *+* |  |  |  | *+* |  | *+* |  |  |  |  |  | *+* |  |  | *+* | *x* | *x* | *v* |
| *YML15* | *+* |  |  |  |  |  | *+* |  |  |  |  |  | *+* | *+* | *+* |  | *x* | *x* | *v* |
| *YML21* | *+* | *+* | *+* |  | *+* |  | *+* | *+* |  |  | *+* | *+* | *+* |  | *+* | *+* | *x* | *x* | *v* |
| *YML22* | *+* | *+* | *+* | *+* | *+* |  | *+* |  |  |  | *+* | *+* | *+* |  |  |  | *x* | *x* | *v* |

Table S1B. Hybrid clones from a cross between *S. cerevisiae* and *S. paradoxus*

| *Clone#* | *Sp-Chromosome* | | | | | | | | | | | | | | | | *Sp-MRS1* |
| --- | --- | --- | --- | --- | --- | --- | --- | --- | --- | --- | --- | --- | --- | --- | --- | --- | --- |
|  | *1* | *2* | *3* | *4* | *5* | *6* | *7* | *8* | *9* | *10* | *11* | *12* | *13* | *14* | *15* | *16* |  |
| *YCL1* | *+* |  | *+* |  |  |  |  |  |  | *+* | *+* | *+* | *+* | *+* |  |  | *v* |
| *YCL2* |  | *+* | *+* |  |  |  | *+* | *+* |  | *+* |  | *+* |  | *+* |  |  | *v* |
| *YCL3* | *+* |  | *+* |  |  | *+* |  |  |  | *+* |  |  |  | *+* |  |  | *v* |
| *YCL4* |  | *+* | *+* |  |  |  |  |  |  | *+* |  |  | *+* | *+* | *+* | *+* | *v* |
| *YCL5* |  |  | *+* |  | *+* |  | *+* |  |  | *+* | *+* |  |  | *+* | *+* | *+* | *v* |
| *YCL6* | *+* | *+* | *+* |  |  |  |  |  |  | *+* | *+* | *+* | *+* | *+* | *+* |  | *v* |
| *YCL7* | *+* |  | *+* |  |  |  |  |  |  | *+* | *+* |  |  |  |  | *+* | *v* |
| *YCL8* | *+* | *+* | *+* |  | *+* |  |  |  |  | *+* |  |  | *+* | *+* |  | *+* | *v* |
| *YCL9* |  |  | *+* |  |  |  |  |  |  |  | *+* |  | *+* | *+* |  |  | *v* |
| *YCL10* | *+* | *+* | *+* |  |  |  | *+* | *+* |  | *+* |  |  |  | *+* | *+* |  | *v* |
| *YCL11* |  | *+* | *+* |  | *+* | *+* |  |  |  | *+* | *+* |  | *+* | *+* |  | *+* | *v* |
| *YCL12* | *+* |  | *+* |  | *+* |  |  |  |  |  | *+* | *+* | *+* | *+* | *+* |  | *v* |
| *YCL13* | *+* | *+* | *+* |  | *+* | *+* |  |  |  |  |  | *+* | *+* | *+* | *+* | *+* | *v* |
| *YCL14* | *+* |  | *+* |  | *+* |  | *+* | *+* |  | *+* | *+* |  |  | *+* | *+* | *+* | *v* |
| *YCL15* | *+* |  | *+* |  | *+* | *+* | *+* |  |  |  | *+* | *+* | *+* |  |  | *+* | *v* |
|  |  |  |  |  |  |  |  |  |  |  |  |  |  |  |  |  |  |
| *YAL11* | *+* |  | *+* |  | *+* |  |  | *+* |  | *nd* | *nd* | *+* | *+* | *+* | *+* |  | *v* |
| *YAL12* | *+* | *+* | *+* |  | *+* | *+* |  | *+* |  | *nd* | *nd* | *+* | *+* | *+* | *+* | *+* | *v* |
| *YAL13* | *+* |  | *+* |  | *+* |  | *+* |  |  | *nd* | *nd* |  |  | *+* | *+* | *+* | *v* |
| *YAL14* | *+* |  | *+* |  | *+* | *+* | *+* |  |  | *nd* | *nd* |  | *+* | *+* |  | *+* | *v* |
| *YAL15* | *+* | *+* | *+* |  |  |  | *+* |  |  | *nd* | *nd* | *+* |  | *+* | *+* | *+* | *v* |
| *YAL16* | *+* |  | *+* |  | *+* |  | *+* |  |  | *nd* | *nd* |  |  | *+* |  |  | *v* |
| *YAL17* | *+* |  |  |  | *+* |  |  |  |  | *nd* | *nd* | *+* |  | *+* |  | *+* | *v* |
| *YAL18* | *+* |  | *+* |  |  |  | *+* |  |  | *nd* | *nd* |  |  | *+* | *+* |  | *v* |
| *YAL19* | *+* |  |  |  |  |  |  | *+* |  | *nd* | *nd* |  | *+* |  |  | *+* | *v* |
| *YAL110* | *+* | *+* | *+* |  |  |  | *+* |  |  | *nd* | *nd* |  | *+* | *+* |  |  | *v* |
|  |  |  |  |  |  |  |  |  |  |  |  |  |  |  |  |  |  |
| *YAL211* | *+* | *+* |  |  |  |  |  |  |  | *+* | *+* |  |  |  | *+* |  | *v* |
| *YAL212* | *+* | *+* | *+* |  | *+* | *+* | *+* |  |  | *+* | *+* |  |  |  | *+* | *+* | *v* |
| *YAL213* | *+* | *+* |  |  |  | *+* |  |  |  | *+* |  |  | *+* |  |  |  | *v* |
| *YAL215* | *+* | *+* | *+* |  | *+* | *+* |  |  |  |  | *+* |  | *+* |  |  | *+* | *v* |
| *YAL216* | *+* | *+* | *+* |  |  | *+* |  | *+* |  | *+* | *+* | *+* | *+* | *+* |  | *+* | *v* |
| *YAL217* | *+* | *+* | *+* |  | *+* | *+* | *+* |  |  | *+* |  |  | *+* |  |  | *+* | *v* |
| *YAL222* | *+* | *+* |  |  | *+* | *+* | *+* | *+* |  | *+* | *+* | *+* | *+* | *+* |  | *+* | *v* |
| *YAL223* | *+* |  |  |  |  | *+* | *+* | *+* |  | *+* |  | *+* | *+* | *+* | *+* | *+* | *v* |
