# Supplementary material for: Multiple Molecular Mechanisms Cause Reproductive Isolation between Three Yeast Species
Source: PLoS Biol. 2010 Jul 20;8(7):e1000432. doi: 10.1371/journal.pbio.1000432 (PMC2907292; doi:10.1371/journal.pbio.1000432)
Supplement: Table S2 — Yeast strains. (0.04 MB DOC) [file pbio.1000432.s006.doc]

| *S. cerevisiae* | |
| --- | --- |
| JYL1127 | *MAT****a*** *ura3-1 his3Δ leu2Δ trp1-1 ade2-1 can1::MFAp-HIS3-MF*α*p-LEU2* |
| JYL1128 | *MAT*α *ura3-1 his3Δ leu2Δ trp1-1 ade2-1 can1::MFAp-HIS3-MF*α*p-LEU2* |
| JYL1159 | *MAT****a*** *met*5*Δ* *ura3Δ* *his3Δ* *leu2Δ can1::MFAp-HIS3-MF*α*p-LEU2 YEL001C::G418::URA3* |
| *S. paradoxus* | |
| JYL1137 | *ho*::HPH *MAT****a*** *ura3 lys2 hisΔ::3xHA leu2::3xHA* |
| JYL1138 | *ho*::HPH *MAT*α *ura3 lys2 hisΔ::3xHA leu2::3xHA* |
| JYL917 | *ho*::KAN *lys2* |
| *S. bayanus* | |
| JYL1030 | *ho*::HPH *MAT*α *ura3* *hisΔ::3xHA leu2::3xHA* *can1::MFAp-HIS3-MF*α*p-LEU2* |
| JYL1031 | *ho*::HPH *MAT****a*** *ura3* *hisΔ::3xHA leu2::3xHA* *can1::MFAp-HIS3-MF*α*p-LEU2* |
| JYL1256 | *ho*::HPH |
